# Supplementary material for: Functional Redundancy in Candida auris Cell Surface Adhesins Crucial for Cell-Cell Interaction and Aggregation
Source: Res Sq. 2024 Mar 22:rs.3.rs-4077218. Preprint. [Version 1] doi: 10.21203/rs.3.rs-4077218/v1 (PMC10984083; doi:10.21203/rs.3.rs-4077218/v1)
Supplement: 1 [file NIHPPrs4077218V1-supplement-1.pdf]

Supplemental Material

Supplemental Figure S1. Probing *C. auris* cell-cell adhesion using single-cell force spectroscopy. AFM setup used for single-cell force spectroscopy experiments. A single live *C. auris* cell was attached to a tipless AFM cantilever previously functionalized with polydopamine. This cell probe was moved toward another single *C. auris* cell immobilized on a polystyrene dish and force-distance curves were recorded, allowing quantification of the intercellular adhesion forces.

Supplemental Figure S2. Evaluation of biofilm formation by the 3 mutant strains generated for the *ALS5* and *SCF1* genes (*Δ*1-*Δ*3). A measurement of the metabolic activity of 24 h biofilms based on values of OD_{490} comparing all generated mutant strains to the wild-type. Boxplots show mean and SEM of $n = 3$ biological replicates, each as an average of 4 technical replicates. Statistical analysis was performed by one-way ANOVA and post-hoc Tukey test with *p*-values representing significant differences. P=1.64×10^{-3}, 1.55×10^{-3}, 2.18×10^{-3}, 2.02×10^{-4}, 3.70×10^{-4}, 3.50×10^{-4}, 4.83×10^{-4}, 5.17×10^{-5}, 3.35×10^{-4}, 3.17×10^{-4}, 4.37×10^{-4}, 4.71×10^{-5} **$0.001 < P \leq 0.01$, ***$P < 0.001$.

Supplemental Figure S3. Comparative evaluation of biofilm formation, aggregation and cell-cell adhesion force by the wild-type AR0382 (aggregative) and AR0387 (non-aggregative) phenotypes. (A) Metabolic activity of 24 h biofilms based on measurements of OD_{490}, optical density. Values are means plus standard errors of the means (error bars). Statistical analysis was performed by an unpaired two-sided t-test. Bar-graphs shows mean and SEM of $n = 3$ biological replicates, each as an average of 4 technical replicates. $P = 2.243\times10^{-5}$. **(B)** Aggregation assays, following vigorous vortexing of cell suspensions comparing cell aggregates of AR0382 and AR0387. Bright-field microscopy (lower panel) of aliquots of cell suspensions demonstrating presence of aggregates of AR0382 cells compared to singly suspended cells of AR0387. (**C**) Measurement of rate of cell sedimentation by absorbance readings of OD_{600} of wild-type strains AR0382 and AR0387 over 2 h following vigorous vortexing. Values represent mean OD and SEM of three technical replicates. **(D)** Single-cell force

spectroscopy of *C. auris* cell-cell adhesion. Adhesion force histograms with representative retraction profiles (inset) obtained for the interaction between AR0382 wild-type cells and the interaction between AR0387 cells*;* 2 representative cell pairs are shown for each strain. **(E)** Adhesion force boxplots depicting n = 6 and n = 4 cell pairs for AR0382 and AR0387 respectively. Statistical analysis was performed by an unpaired two-sided t-test. $P = 4.21\times10^{-2}$ **(F)** As in **(E)**, adhesion frequency boxplots show interactions between n = 7 cell pairs for both strains. $P = 8.06\times10^{-6}$. Red stars represent the mean values, red lines are the medians, boxes are the 25−75% quartiles and whiskers the standard deviation from mean. $^{*}0.01 < P \leq 0.05$, $^{***}P < 0.001$.

Table S1. Primers used in this study

Table S2. Differentially expressed genes between AR0382/AR0387 during *in vitro* biofilm growth (FDR <0.01, LFC >= |1.0|)

Table S3. Differentially expressed genes between AR0382/AR0387 during *in vivo* biofilm growth (FDR <0.01, LFC >= |1.0|)

Table S4. List of genes that are more highly expressed in AR382 under both *in vitro* and *in vivo* biofilm conditions (FDR <0.01, LFC >= |1.0|)
